# An Exploratory Study Using Next-Generation Sequencing to Identify Prothrombotic Variants in Patients with Cerebral Vein Thrombosis

**DOI:** 10.3390/ijms24097976

**Published:** 2023-04-28

**Authors:** Robert Anton Kramer, Robert Zimmermann, Julian Strobel, Susanne Achenbach, Armin Michael Ströbel, Holger Hackstein, David Alexander Christian Messerer, Sabine Schneider

**Affiliations:** 1 Department of Transfusion Medicine and Hemostaseology, Friedrich-Alexander University Erlangen-Nuremberg, University Hospital Erlangen, 91054 Erlangen, Germany; robert.kramer@fau.de (R.A.K.); robert.zimmermann@uk-erlangen.de (R.Z.); julian.strobel@uk-erlangen.de (J.S.); susanne.achenbach@uk-erlangen.de (S.A.); holger.hackstein@uk-erlangen.de (H.H.); 2 Center for Clinical Studies (CCS), Medical Faculty, Friedrich-Alexander University Erlangen-Nuremberg, University Hospital Erlangen, 91054 Erlangen, Germany; armin.stroebel@uk-erlangen.de

**Keywords:** cerebral vein thrombosis, thrombophilia, personalized medicine, next-generation sequencing, molecular medicine, neurology, hemostaseology

## Abstract

Prothrombotic hereditary risk factors for cerebral vein thrombosis (CVT) are of clinical interest to better understand the underlying pathophysiology and stratify patients for the risk of recurrence. This study explores prothrombotic risk factors in CVT patients. An initial screening in patients of the outpatient clinic of the Department of Transfusion Medicine and Hemostaseology of the University Hospital Erlangen, Germany, revealed 183 patients with a history of CVT. An initial screening identified a number of common prothrombic risk factors, including Factor V Leiden (rs6025) and Prothrombin G20210A (rs1799963). All patients without relevant findings (58 individuals) were invited to participate in a subsequent genetic analysis of 55 relevant genes using next-generation sequencing (NGS). Three intron variants (*ADAMTS13*: rs28446901, *FN1*: rs56380797, rs35343655) were identified to occur with a significantly higher frequency in the CVT patient cohort compared to the general European population. Furthermore, the combined prevalence of at least two of four potentially prothrombic variants (*FGA* (rs6050), *F13A1* (rs5985), *ITGB3* (rs5918), and *PROCR* (rs867186)) was significantly higher in the CVT subjects. The possible impact of the identified variants on CVT is discussed.

## 1. Introduction

Cerebral vein thrombosis (CVT) is a leading cause of stroke in young adults. In general, it is responsible for approximately 0.5% of all strokes [1]. CVT affects individuals with a mean age of approximately 40 years and has a two-thirds to three-quarters female preponderance [2,3,4]. Common symptoms include minor to life-threatening features such as headache, focal neurological deficits, and seizures [1,2]. The outcome of CVT varies, resulting in approximately 57% of patients lacking symptoms, 35% of patients experiencing minor to severe impairment, and a lethal outcome for 8% of patients [5].

The etiology of CVT can include a complex imbalance of prothrombotic and fibrinolytic processes, including alterations in blood stasis and irregularities in vessel walls and blood composition [2]. The corresponding treatment can briefly be summarized as the initiation of anticoagulation, often initially with low-molecular-weight heparin and, if necessary, treatment of the respective underlying triggers such as dehydration, sepsis, or the cessation of prothrombotic medication [2,6,7].

The risk of CVT recurrence is around 5% per year [2,5]. In this context, the current guidelines recommend anticoagulation with a vitamin K antagonist for three to twelve months, but the recommendation is listed as weak and supported by evidence of very low quality [2,7]. In the case of recurrent CVT, prolonged or lifelong anticoagulation is discussed, but further investigation is still needed [2,8]. Likewise, there is only very limited evidence that screening for hereditary thrombophilia prevents recurrent venous thrombosis in patients with CVT [6,7]. Nevertheless, several hereditary risk factors for CVT have been identified. For example, protein C and protein S deficiency have odds ratios of 11.1 and 12.5, respectively (see Table 3 in [6]). However, the standard screening for thrombophilia might miss certain rare, hereditary thrombophilias, which is a matter of further investigation [4,7,9].

From a clinical perspective, the clarification of a potential hereditary thrombophilia contributes to the balance between anticoagulation and bleeding risk when discussing an individual long-term anticoagulation strategy. In this context, contemporary technologies, such as next-generation sequencing (NGS), enable the clinician to further recognize individual risk factors [10,11]. However, these approaches require a resource-intensive evaluation of the patient, including high personal costs for consulting and the interpretation of the results and the corresponding equipment and reagents. This complex situation is further underlined by the fact that current guidelines do not comment on elaborate genetic analysis, likely due to the lack of available data on a possible improvement in the treatment and outcome of CVT patients [2,6,7].

Therefore, the present study investigates CVT patients who presented at an outpatient university clinic for hemostaseological follow-up. The current work (1) reports on the findings of an initial standard thrombophilia screening that included Factor V Leiden mutation, Prothrombin G20210A mutation, protein C and protein S deficiencies, antithrombin III deficiency, and antiphospholipid syndrome; and (2) explores the value of advanced molecular genetic analysis in patients with CVT without conclusive results in a basic thrombophilia screening.

## 2. Results

### 2.1. Initial Thrombophilia Screening

**Figure 1:**
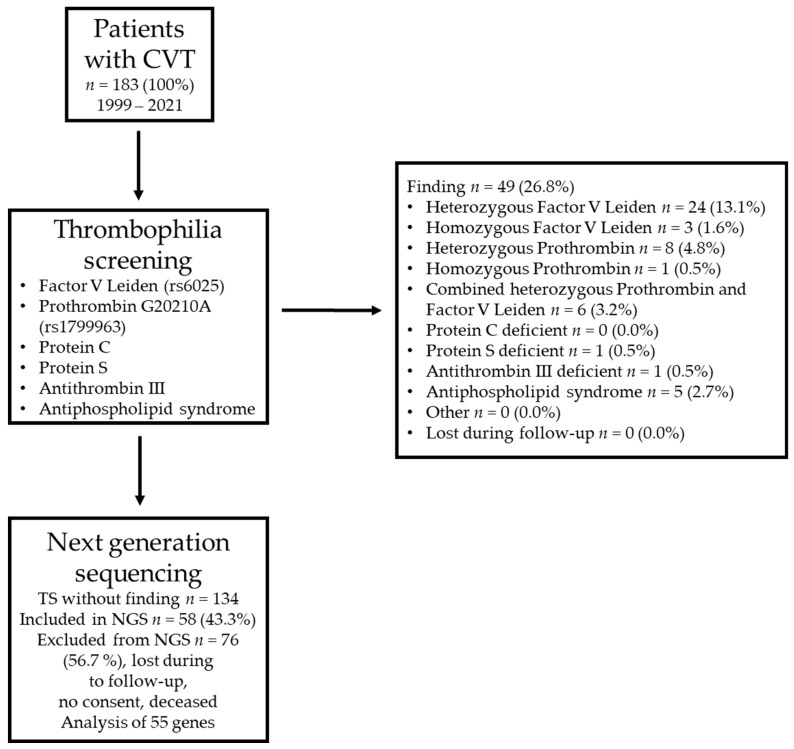
Summary of the cohort and the stepwise approach to identifying patients with cerebral vein thrombosis (CVT) and negative results in a basic screening for thrombophilia. TS = thrombophilia screening. The 55 analyzed genes are listed in Supplemental Table S1. NGS = next-generation sequencing.

The stepwise diagnostic approach of this study is summarized in Figure 1. The cohort consisted of 33 males and 150 females. The initial thrombophilia screening included 183 patients and resulted in 49 (26.8%) positive findings. The heterozygous Factor V Leiden mutation (13.1%) was the most prevalent risk factor for thrombophilia, followed by the heterozygous Prothrombin G20210A mutation (4.8%) and the combined heterozygous Prothrombin G20210A and Factor V Leiden mutation (3.2%). For 73.2% of the patients, the initial screening for thrombophilia did not reveal a relevant finding (Figure 1). Although female patients (*n* = 150) were more numerous compared to male patients (*n* = 33) within the population, the proportion in frequency of common hereditary thrombophilia was almost the same (female: 26.7%, male: 27.3%, *p* > 0.99; Fisher’s exact test). 

### 2.2. Analysis of NGS Data

**Figure 2:**
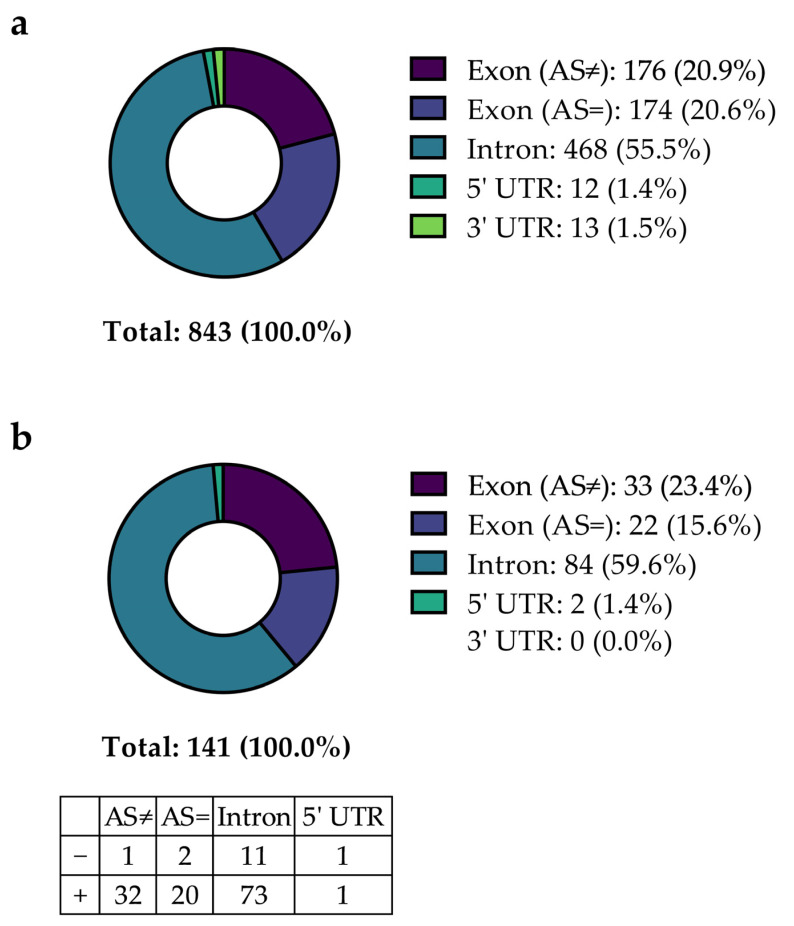
Summary of variants detected within the study population of patients with CVT with initial negative thrombophilia screening. Categorization of the reported significant variants without (**a**) or with (**b**) correction for multiple testing, including mutations in introns, exons (with (AS≠) or without (AS=) impact on amino acid configuration), and untranslated regions (5′ UTR), and additionally split according to a higher or lower prevalence compared to the prevalence reported for a general European population by the NCBI, respectively (table inset in (**b**)).

As a second step, all patients without hits in the first screening for thrombophilia were invited to participate in the study, including NGS. Out of 134 patients, 58 (43.3%) were included in this study. The NGS approach contained the sequencing of 55 hemostasiologically relevant genes (complete coding sequences and adjacent intron regions) (Supplemental Table S1). In total, 843 variants were identified, including common polymorphisms and rare mutations (Figure 2a). A total of 468 variants were detected in introns (55.5%), and 350 variants were detected in exon regions with (176 (20.9%)) or without (174 (20.6%)) a change in the encoded amino acid. Twelve (1.4%) variants were found in the five prime untranslated region (5′ UTR), and thirteen (1.5%) were found in the three prime untranslated region (3′ UTR).

When comparing the prevalence of the 843 identified variants within the study population with the general European population (data taken from the NCBI dbSNP database), 141 variants (16.7%) showed a statistically significant distribution discrepancy (unadjusted *p* < 0.05) (Figure 2). Figure 2b describes the distribution of these 141 variants identified depending on their localization. The most significant variants were detected in intronic regions (84 of 141 (59.6%)), followed by variants in exons with (33 (23.4%)) or without (22 (15.6%)) affecting the respective amino acid. Two (1.4%) variants were found in the 5′ UTR, and none were found in the 3′ UTR. Most variants (89.4%) were more prevalent in the SVT population compared to the frequency reported by the NCBI, while 10.6% were less prevalent. Based on a significantly different prevalence, these variants were considered to be potentially involved in favor of thrombophilia and thus to be possibly associated with CVT. Normalizing the identified significant variants on 1 kilobase (1 kb) gene lengths, most variants were identified in genes that encode Protein Z (*PROZ*), Neurobeachin-like 2 (*NBEAL2*), and Filamin A (*FLNA*) (Supplemental Figure S1). Differences in variation counts per gene were expected because the variation frequency per gene depends on multiple factors, such as the position on the chromosome, the rate of recombination of the respective chromosomal region, and tolerance to mutations [12].

**Figure 3:**
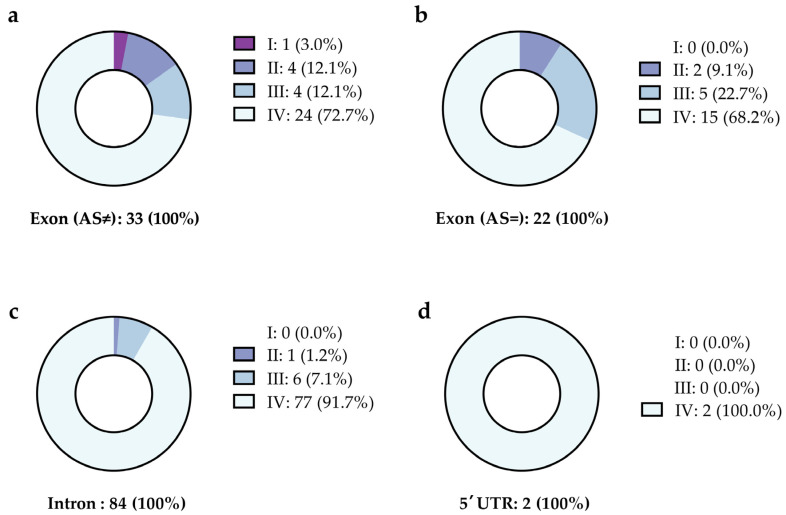
Categorization of variants with a significantly different allele frequency in patients with a history of CVT and a negative initial screening for thrombophilia. The variants are listed depending on their location and are reported as (**a**) exon with exchange of the amino acid, (**b**) exon without exchange in the amino acid, (**c**) intron, and (**d**) the five prime untranslated region (5′ UTR). Figure 3 summarizes the data presented in Supplemental Table S2. The variants were classified as (I) prothrombotic, (II) probably prothrombotic, (III) mentioned in the context of other diseases, and (IV) unknown, as explained in the methods section.

The reported variants were categorized as described in the Methods section to briefly estimate their relevance (Figure 3). For most variants, no information was available, according to the NCBI database.

For 34 of the 141 variants, the frequency of the alternate allele provided by the NCBI is zero, indicating that these 34 variants might be rare mutations (indicated in gray letters in Supplemental Table S2). No further information on any of these variants was available. By correcting the *p* values for multiple tests by multiplying the resulting *p* value with the number of tests, 28 of the remaining 107 variants remained significant, with a *p* value < 0.05 (highlighted by a gray background in Supplemental Table S2). To exclude that the variation in allele frequency is due to bias (rare mutations found by chance), the focus was placed on those variants with a prevalence of more than 5% (number of alternative alleles > 6). This cutoff was chosen with respect to the prevalence of the Prothrombin G20210A mutation (4.8%). This cutoff identified two intron variants in the *FN1* gene (rs56380797 and rs35343655) and one *ADAMTS13* intron variant (rs28446901) (highlighted by a black background and white letters in Supplemental Table S2). A potential prothrombotic impact of these variants is discussed below.

As certain variants appear frequently in the screening for a genetic predisposition to thrombophilia in the authors’ department, the combined presence of two out of four representative variants was analyzed. The variants chosen for the combination analysis were rs6050 (*FGA*: c.991A>G; p.Thr331Ala), rs5985 (*F13A1*: c.103G>T; p.Val35Leu), rs5918 (*ITGB3*: c.176T>C; p.Leu59Pro), and rs867186 (*PROCR*: c.655A>G; p.Ser219Gly). These variants are localized on different autosomal chromosomes; thus, the genetic linkage of these variants and sex-dependent inheritance could be excluded. The potential prevalence of each combination in the general European population or in the study group was calculated by multiplying the relative allele frequencies deposited in the NCBI database or collected in this study, respectively. Subsequently, the calculated prevalence was compared with the actual prevalence obtained in this study (Table 1). Interestingly, not only was the calculated prevalence of each combination higher in our study compared to the general European population but the actual prevalence of each combination exceeded the calculated values. The Chi-squared test revealed a significant difference (*p* < 0.05) between the actual frequency determined in the CVT cohort compared to the calculated frequency in the general European population for all combinations except *PROCR* (rs867186) + *ITGB3* (rs5918). A combination of at least two of the respective variants was found in 29 of 58 study participants (50.0%).

**Figure 4:**
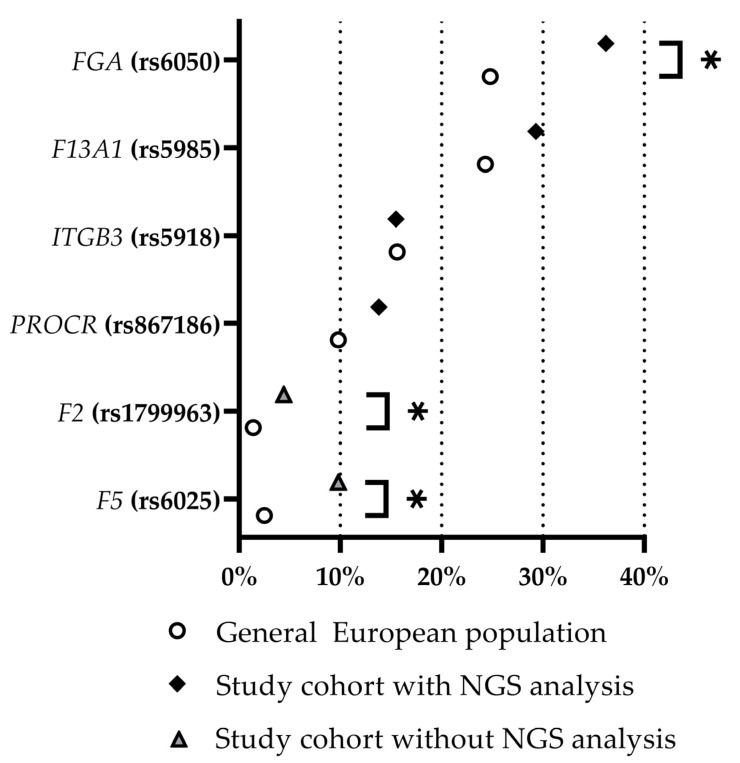
Relative frequency of identified prothrombotic variants within the study cohort of CVT patients (with and without NGS analysis, as indicated) and within the general European population (based on NCBI data). Asterisks mark significantly different frequencies (*p* value < 0.05 without correction for multiple testing). NGS = next-generation sequencing. Prothrombin G20210A mutation and Factor V Leiden mutation are indicated as *F2* (rs1799963) and *F5* (rs6025), respectively.

The variants chosen for the combination analysis did not attract attention during the first evaluation as their prevalence in the CVT cohort did not differ significantly from the prevalence deposited in the NCBI from the very beginning (this applies to rs5985 (*F13A1*), rs5918 (*ITGB3*), and rs867186 (*PROCR*)) or after multiple testing correction (rs6050 (*FGA*)). After analyzing the combined prevalence of these variants, their allele frequency within the study group with NGS analysis compared to the general European population was reevaluated. The prothrombotic variants (Prothrombin G20210A mutation and Factor V Leiden mutation) of the patients in the study group that did not undergo sequencing were included. Figure 4 summarizes the results of this analysis. Except for the *ITGB3* variant rs5918, all other variants showed a higher prevalence within the cohort of patients suffering from CVT, with CVT/general ratios ranging from 1.0 (*ITGB3*; rs5918) to 3.9 (*F5*; rs6025). A significant difference according to a test for an analysis of proportions with a *p* value < 0.01 and without correction for multiple testing is provided for the Factor V Leiden, Prothrombin G20210A, and rs6050 (*FGA*) variants. After correction for multiple testing, a *p* value < 0.01 remained for Factor V Leiden and Prothrombin G20210A. Among the 58 CVT patients, one out of the four variants (rs6050 (*FGA*), rs5985 (*F13A1*), rs5918 (*ITGB3*) and rs867186 (*PROCR*)) was found in 21 subjects (36.2%), and two or more were found in 29 subjects (50.0%). None of the four variants were found in eight subjects (13.8%).

## 3. Discussion

The present study evaluated a stepwise approach to diagnosing prothrombotic variants in patients with a history of CVT. This monocentric study focused on patients who presented at a university outpatient clinic in Erlangen, Bavaria, Germany. Due to the low prevalence of CVT, all patients were included if electronic files were available. Although this increased the sample size compared to other CVT studies [13], several clinical data points, such as age at the time of CVT diagnosis, coincidence with cancer, etc., were not available. However, the distribution of sexes in the collective in the present study was comparable to CVT in general [2,4]. Based on the recruitment strategy, a certain bias due to the monocentric nature of the study and due to nonparticipation (for example, due to lethality) cannot be excluded.

In the initial screening for thrombophilia, the heterozygous Factor V Leiden mutation was the most prevalent prothrombotic risk factor, which is in accordance with the corresponding literature [13,14]. In general, approximately one out of four patients with a history of CVT were positive for a common prothrombotic variant in the initial screening for thrombophilia. The additional genetic analysis of patients without a common prothrombotic variant revealed (1) a significantly higher frequency of the intronic variants rs56380797, rs35343655 (both *FN1*), and rs28446901 (*ADAMTS13*); and (2) a noticeable accumulation of the presence of two prothrombic variants of the *FGA*, *F13A1*, *ITGB3,* or *PROCR* gene, respectively, in combination.

The *ADAMTS13* gene encodes a metalloprotease that specifically cleaves von Willebrand factor multimers. Compound heterozygous or homozygous mutations in the *ADAMTS13* gene are associated with hereditary thrombotic thrombocytopenic purpura (TTP, also known as Upshaw–Schulman syndrome). TTP manifests itself in severe thrombocytopenia, microangiopathic hemolytic anemia, and organ ischemia due to disseminated platelet-rich thrombi in the microvasculature but not in large vessels [15,16,17]. Thus, although the frequency of rs28446901 in *ADAMTS13* differs significantly in the study cohort compared to the general European population, a clinical impact concerning CVT seems rather unlikely.

Fibronectin (encoded by *FN1*) is a multifunctional structural protein of the extracellular matrix that occurs as plasma fibronectin or cellular fibronectin, depending on the splicing variant [18]. Fibronectin plays a role in regulating platelet thrombus formation and is a structural part of the blood clot after being integrated into the fibrin network by factor XIII [18,19]. Elevated plasma fibronectin levels were shown to be associated with venous thromboembolism [20]. Based on alternative *FN1* mRNA splicing, 20 different transcript variants have been described to date [21]. Eighteen transcript variants (according to availability in NCBI) were analyzed using VarSEAK for potentially different splicing caused by the *FN1* variants rs56380797 or rs35343655. According to an in silico analysis, splicing is not affected for any isoform by the *FN1* variants rs56380797 or rs35343655.

Interestingly, the *FN1* variant rs56380797 (c.3253+66C>A) is, with respect to the data obtained in our study cohort, inherited together with the variants rs13306359 (c.3253+17G>A), rs7589580 (c.3111A>C; p.Gly1037=), and rs1053238 (c.3156A>C; p.Pro1052=). No further information is available for any of these variants, and a potential effect on splicing of the variant rs13306359 was excluded after in silico analysis with VarSEAK. Nevertheless, an impact of the *FN1* intron variants rs56380797 and rs35343655 alone or the simultaneous occurrence of the variants rs56380797, rs13306359, rs7589580, and rs1053238 on fibronectin levels is in the range of possibility, as intron variants and synonymous variants are described to affect gene expression and protein production in multifold manners [22,23]. A potential association of the *FN1* intron variants with CVT is, according to the current knowledge, highly speculative. Further examination, for example, by quantifying fibronectin levels in the study cohort, is vital in proving a possible connection between the *FN1* variants rs56380797 and/or rs35343655 and CVT.

During the evaluation of the NGS data, a remarkably high number of subjects were found to carry at least two of the four potentially prothrombic variants rs6050 (*FGA*), rs5985 (*F13A1*), rs5918 (*ITGB3*), and rs867186 (*PROCR*).

The SNP rs6050 in the *FGA* gene (which encodes the fibrinogen α-chain) has been under consideration as a potential prothrombic factor for decades [24,25,26,27,28,29]. The amino acid exchange p.Thr331Ala results in increased factor XIII cross-linking and thicker fibrin fibers, producing stiffer clots which are likely more susceptible to embolization [26].

*F13A1* encodes the A chain, which is the catalytic active subunit of factor XIII. At the molecular level, Val35Leu factor XIII is activated more rapidly by thrombin and alters the fibrin structure in terms of fiber diameter and permeation characteristics, with a shorter clot formation time and more compact fibrin clots that are less susceptible to lysis [30,31,32]. The association of the amino acid exchange p.Val35Leu (rs5985) with thromboembolic events (also in conjunction with the *FGA* variant rs6050) is a matter of debate [25,28,33,34,35,36,37,38,39].

Although some studies reported a protective impact of *F13A1*, at least under distinct conditions such as increased fibrinogen levels or only in the presence of homozygous Thr331 in the fibrinogen α-chain [25,28,33,34,35], others do not describe a protective influence [30,36,37]. In contrast, some studies claimed an association of Val35Leu with an increased risk for pulmonary embolism [30,38] or a predisposition to the development of severe post-thrombotic syndrome [39]. Interestingly, a case report of a CVT patient described the homozygous presence of Val35Leu as a potential additional risk factor for thrombophilia [40]. Taken together, due to the changes described for the structure of the blood clot generated by Ala331 in the fibrinogen α-chain and Leu35 in the factor XIII A chain as well as the higher abundance of a combined presence of both minor alleles in the CVT cohort of this study, an increased prothrombic risk for carriers of both minor alleles seems likely.

*ITGB3* encodes the β3 subunit of integrin αIIbβ3, which mediates platelet adhesion to fibrin, the von Willebrand factor, and fibronectin [41]. Regarding *ITGB3* (rs5918), it has been demonstrated that the amino acid exchange Leu59Pro results in enhanced fibrinogen binding, which leads to a higher aggregation of platelets, suggesting this gain-of-function variant is a prothrombic risk factor [42,43,44,45]. Mechanically induced changes in fibrin structure and fibrin density were shown to alter the binding of integrin αIIbβ3 [46,47]. Therefore, an additionally enhanced binding of integrin β3 Leu59Pro to a fibrin structure modified by *F13A1* Val35Leu and *FGA* Thr331Ala is speculative but not unlikely.

The endothelial protein C receptor (EPCR; encoded by *PROCR*) is, in principle, membrane-bound; however, it is also present in a soluble form (sEPCR) to a certain extent. The amino acid exchange Ser219Gly (rs867186) shifts the balance between membrane-bound and soluble to higher levels of sEPCR [48,49,50,51,52,53]. Increased levels of sEPCR are supposed to result in reduced activation of protein C which, in turn, is considered to increase the risk of thrombosis [49,52,53,54,55,56,57]. A reduction in the profibrinolytic effect of activated protein C, induced by *PROCR* rs867186, in combination with variants that alter the fibrin structure towards an increased lysis resistance and are therefore prothombic might constitute a relevant risk factor for thrombophilia. Therefore, an amplifying effect of two prothrombic variants could be postulated for all combinations except *PROCR* (rs867186) + *ITGB3* (rs5918). Interestingly, this combination is the only one that shows no statistically significant difference in frequency compared to the general European population. Likewise, frequencies of only one out of the four respective variants do not significantly differ (after correction for multiple testing) compared to the general European population (Figure 4), suggesting that it is the combination of variants that increases the risk of thromboembolic events.

Several studies postulated a combinatory effect of prothrombotic variants on thromboembolic events [58,59,60,61] and for rare thrombotic manifestations such as central retinal vein occlusion [62]. Nevertheless, the in vivo effect of the simultaneous presence of combinations of variants rs6050 (*FGA*), rs5985 (*F13A1*), rs5918 (*ITGB3*), and rs867186 (*PROCR*) remains to be elucidated on a molecular level. This could be conducted, for example, in a model system and/or by a subsequent confirmatory study with a higher number of patients that also includes a cohort of healthy subjects for a direct comparison.

Thus far, in the study cohort of CVT patients, well-known risk factors for thrombophilia and a possible combinatory impact of potentially prothrombic variants were found. No specific mutation was identified that could unambiguously be assigned to the defined clinical picture of CVT and not to the general risk of thrombophilia. This is probably due to the complex coagulation cascade and the fact that the 55 genes analyzed in this study cover only a limited range of the multifaceted processes that might lead to CVT. A whole-exome study that includes, for example, genes that promote the development of cerebral veins and those that encode proteins essential for structural integrity (e.g., extracellular matrix proteins that build the basal lamina, such as collagen type IV, laminin, heparin sulfate, proteoglycans, and others [63]), will further elucidate this matter.

However, the sequencing of the selected 55 genes to support routine diagnostics for thrombophilia patients provides useful information to unravel potential hereditary thrombotic risk factors. In this context, it is essential to mention that the effects of rs6050 (*FGA*), rs5985 (*F13A1*), rs5918 (*ITGB3*), and rs867186 (*PROCR*) on their respective proteins cannot be depicted by standard laboratory diagnostics so far, making identification at the DNA level necessary. By including an NGS analysis, an incomparably larger amount of information is available, including not only the four prothrombic variants focused on in this study but also other potentially prothrombic variants that can only be identified at the genetic level, for example, the *GP1BA* variant rs2243093 [64,65,66] or the *VWF* variant rs35335161 [67]. Therefore, the NGS approach is a valuable tool for supporting standard diagnostic procedures for patients with complex thromboembolic events.

There are several limitations to this study. First, the number of subjects was relatively small, which can be attributed to the fact that CVT is a rather rare thrombotic event. Second, for economic reasons, this study did not include age- and sex-matched healthy volunteers of the same age and sex in the NGS approach. Third, a selection of 55 genes was analyzed. The selected genes cover proteins involved in the most important processes of coagulation. However, variations in additionally relevant genes that were not analyzed might be causally related to the development of CVT.

## 4. Materials and Methods

### 4.1. Patient Cohort and Sampling Approach

Ethical approval was granted by the local independent ethics committee of Friedrich-Alexander University Erlangen-Nuremberg, Erlangen, Germany (#162_21 B and 21-162_1-B). The study (and hemostaseological counseling in general) consisted of a stepwise approach. In an initial retrospective screening, all patients with a history of CVT who presented at the Department of Transfusion Medicine and Hemostaseology of the University Hospital Erlangen during the period of 1999–2021 were included. Prior to 1999, no hemostaseological outpatient clinic existed in the department. Overall, 183 patients (150 females; 33 males) with CVT presented in this period (out of 22,902 patient encounters).

Next, patients with CVT were selected for eligibility to be included in the second part of the study, which included the NGS approach, as described below. Patients with a positive result in the basic screening for thrombophilia were excluded.

### 4.2. Initial Thrombophilia Screening

Initially, all CVT patients received a basic screening for thrombophilia in the clinically accredited hemostaseologic routine laboratory of the Department of Transfusion Medicine and Hemostaseology of the University Hospital Erlangen. The methods of the screening parameters, but not the prothrombotic factors per se, changed in the observation period. The latest analyses were conducted as subsequently listed: Factor V Leiden mutation (rs6025) (FluoroType Factor V, Hain Lifescience GmbH, Nehren, Germany), Prothrombin G20210A mutation (rs1799963) (FluoroType Factor II, Hain Lifescience GmbH, Nehren, Germany), protein C deficiency (STACHROM Protein C, STAGO, Asnières-sur-Seine, France), protein S deficiency (STAGO, chromogenic procedure), antithrombin III deficiency (STAGO, chromogenic procedure), and antiphospholipid syndrome (AESKULISA ß2-Glyco-GM is a solid-phase enzyme immunoassay for the quantitative and qualitative detection of IgG and/or IgM antibodies against ß2 glycoprotein I in human serum, and AESKULISA Cardiolipin-GM is a solid-phase enzyme immunoassay for the quantitative and qualitative detection of IgG and/or IgM antibodies against cardiolipin in human serum) (Figure 1).

### 4.3. NGS

All patients with a history of CVT who presented between 1999 and 2021 at the outpatient clinics of the department without conclusive findings in the initial screening for thrombophilia as described above were invited to participate in this study (Figure 1). After informed written consent was obtained, blood was drawn, and the genomic DNA was automatically isolated using commercial kits (NucleoSpin 8 Blood Core Kit, Macherey Nagel, Düren, Germany) and a Hamilton Microlab Starlet robot (Reno, NV, USA). Genomic DNA was quantified using the Hamilton Microlab Starlet robot (Reno, NV, USA) and a commercial kit (QuantiFluor dsDNA System, Promega, Madison, WI, USA).

For the NGS analysis of the genes of interest, an Illumina amplicon-based gene panel (AmpliSeq Custom DNA Panel for Illumina) and AmpliSeq Library PLUS kit (Illumina, San Diego, CA, USA) were used for library generation according to the manufacturer’s instructions. The genes of interest were selected according to the work of Simeoni et al. [10], with modifications. The sequencing of the obtained libraries was performed on a MiSeq machine (Illumina, San Diego, CA, USA). The custom DNA panel was manufactured according to the department’s requirements and allows for the sequencing of the coding sequences and adjacent intron regions of the following genes: *ACTN1*, *ACVRL1*, *ADAMTS13*, *ANKRD26*, *CPB2*, *CYCS*, *ENG*, *F2*, *F5*, *F7*, *F8*, *F9*, *F10*, *F11*, *F13A1*, *F13B*, *FGA*, *FGB*, *FGG*, *FLNA*, *FN1*, *GGCX*, *GP1BA*, *GP1BB*, *GP6*, *GP9*, *HRG*, *ITGA2B*, *ITGB3*, *KLKB1*, *KNG1*, *LMAN1*, *MCFD2*, *MPL*, *MYH9*, *NBEAL2*, *P2RY12*, *PLA2G4A*, *PLAU*, *PLG*, *PROC*, *PROCR*, *PROS1*, *PROZ*, *SERPINC1*, *SERPINE1*, *SLC44A2*, *TBXAS1*, *TBXA2R*, *THBD*, *THPO*, *TUBB1*, *VKORC1*, *VWF*, *WAS* (Supplemental Table S1). The sequences obtained were analyzed using SeqNext software version 5.2.0 (JSI Medical Systems, Ettenheim, Germany).

### 4.4. Data Analysis

The resulting allele frequencies in the primary thrombophilia screening and the NGS approach were compared to the frequency of their respective variants in the European population, as reported by the Single Nucleotide Polymorphism Database (dbSNP) of the National Center for Biotechnology Information (hereafter referred to as NCBI; Bethesda, MD, USA) of the United States of America (last accessed: 10 March 2023) [68]. To calculate the combination of certain variants, a random distribution of the respective variants was assumed. VarSeak online (https://varseak.bio/, accessed on 28 March 2023; JSI medical systems) was used to briefly analyze the potential impact of variants on mRNA splicing in silico. The identified variants were further grouped into four categories: category I refers to variants that have been described in the literature as likely general prothrombotic risk factors; category II summarizes variants that have been discussed in the context of thrombophilia; category III consists of variants that have been reported in conjunction with other diseases and without a link to thrombophilia; and category IV lists variants without further information, according to NCBI.

All data were analyzed using Excel 2016 (Office Professional Plus 2016) (Microsoft, Redmond, WA, USA), Prism 9 (version 9.4.2, GraphPad Software, Boston, MA, USA), and R 4.2.1 [69]. To compare the prevalence of the identified variants in the study population with a general population, the NCBI database of Genotypes and Phenotypes Release 2 was accessed. The frequencies of the variants of interest were compared between the study population and the European population, using a test for proportions implemented in the R-function prop test [70]. When indicated, the *p* values were corrected for multiple tests by multiplying the resulting *p* value by the number of tests. If not indicated otherwise, data are provided as medians with an interquartile range. Categorial variables were analyzed using Fisher’s exact test. The *p* values in Table 1 were generated using the Chi-squared test.

## 5. Conclusions

This monocentric study explored the value of a two-step approach to assessing patients with a history of CVT with respect to prothrombotic variants. Including NGS in the diagnostic workup identified certain variants of interest that affect almost all patients with a history of CVT. Further large cohort studies are needed to confirm the hypotheses based on the results of this study.

## Figures and Tables

**Table 1:** Combined prevalence of representative variants of interests. The actual frequency in CVT patients (Act. Freq. CVT) refers to the measured numbers in the study group. Calculated frequencies (Calc. Freq.) refer to the estimated prevalence, assuming random distribution in the study population (patients with CVT, *n* = 116) and the general European population (NCBI, *n* = 38,782–296,470), respectively. The *p* values were calculated comparing the Act. Freq. CVT vs. Calc. Freq. NCBI. Chi-squared test was used for the calculation of *p* values; *p* values were not corrected for multiple tests due to the low number of analyses.

Variants	Act. Freq. CVT (%)	Calc. Freq. CVT (%)	Calc. Freq. NCBI (%)	*p* Value
*FGA* (rs6050) + *F13A1* (rs5985)	15.52	10.61	6.03	<0.001
*FGA* (rs6050) + *ITGB3* (rs5918)	9.48	5.62	3.87	0.004
*FGA* (rs6050) + *PROCR* (rs867186)	7.76	4.99	2.44	0.001
*ITGB3* (rs5918) + *F13A1* (rs5985)	11.21	4.55	3.81	0.001
*PROCR* (rs867186) + *F13A1* (rs5985)	5.17	4.04	2.39	0.024
*PROCR* (rs867186) + *ITGB3* (rs5918)	2.59	2.14	1.54	0.59

## Data Availability

All relevant data are included in the manuscript. Further original data will be made available by contacting the corresponding authors within the regulations of the ethical approval.

## Supplementary Materials

The following supporting information can be downloaded at: https://www.mdpi.com/article/10.3390/ijms24097976/s1.
