# Supplementary material for: An Exploratory Study Using Next-Generation Sequencing to Identify Prothrombotic Variants in Patients with Cerebral Vein Thrombosis
Source: Int J Mol Sci. 2023 Apr 28;24(9):7976. doi: 10.3390/ijms24097976 (PMC10178986; doi:10.3390/ijms24097976)
Supplement: Supplementary file 1 [file ijms-24-07976-s001.zip › ijms-2369719-supplementary.pdf]

International Journal of
Molecular Sciences

Supplement

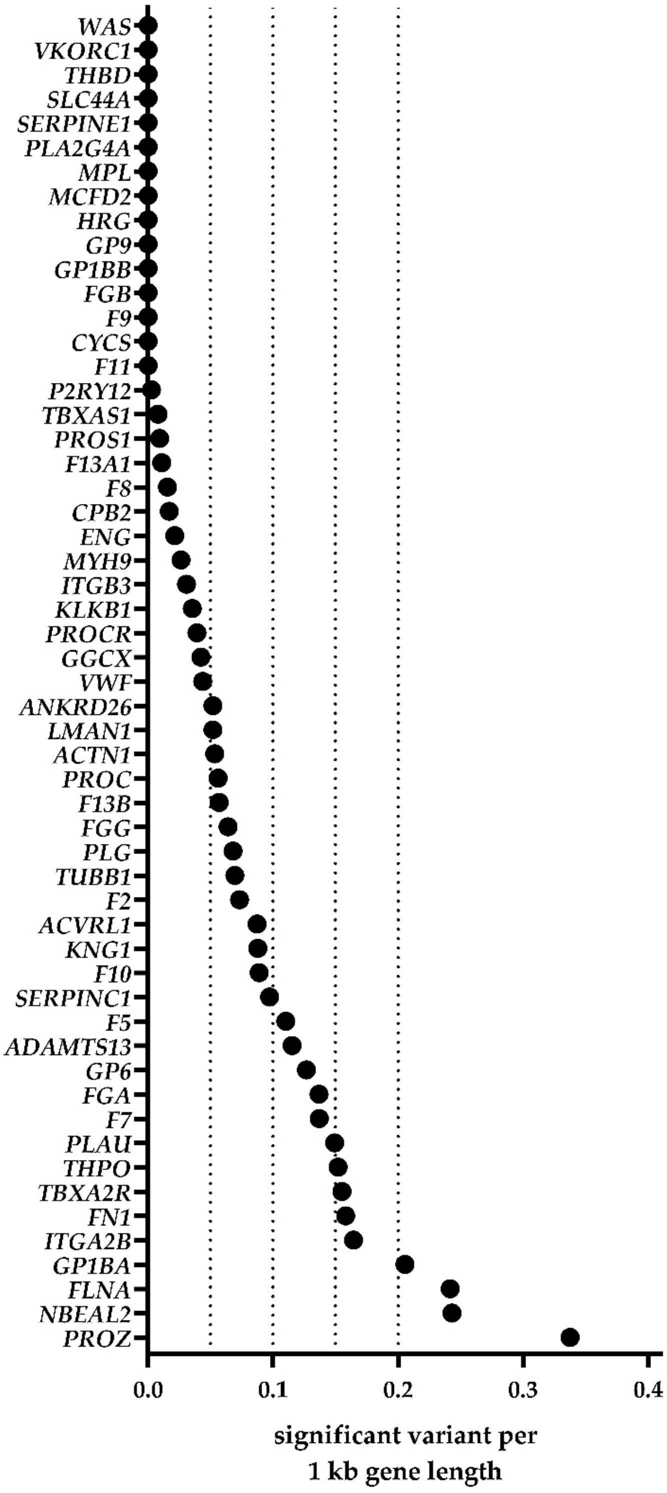

Supplemental Figure S1. Frequency of significant variants per 1 kb (kilobase) gene length within the listed gene.

Supplemental Table S1. Summary of the 55 genes included in the NGS approach.

Gene	Protein	Position
ACTN1	Actinin Alpha 1	Chr. 14
ACVRL1	Activin A Receptor Like Type 1	Chr. 12
ADAMTS13	A Disintegrin and Metalloproteinase with Thrombospondin Type 1 Motif 13	Chr. 9
ANKRD26	Ankyrin Repeat Domain Containing 26	Chr. 10
CPB2	Carboxypeptidase B2 (= TAFI, Thrombin Activatable Fibrinolysis Inhibitor)	Chr. 13
CYCS	Cytochrome C, Somatic	Chr. 7
ENG	Endoglin	Chr. 9
F2	Coagulation Factor II, Prothrombin	Chr. 11
F5	Coagulation Factor V	Chr. 1
F7	Coagulation Factor VII	Chr. 13
F8	Coagulation Factor VIII	Chr. X
F9	Coagulation Factor IX	Chr. X
F10	Coagulation Factor X	Chr. 13
F11	Coagulation Factor XI	Chr. 4
F13A1	Coagulation Factor XIII, A Chain	Chr. 6
F13B	Coagulation Factor XIII, B Chain	Chr. 1
FGA	Fibrinogen, alpha Chain	Chr. 4
FGB	Fibrinogen, beta Chain	Chr. 4
FGG	Fibrinogen, gamma Chain	Chr. 4
FLNA	Filamin A	Chr. X
FN1	Fibronectin 1	Chr. 2
GGCX	Gamma-Glutamyl Carboxylase	Chr. 2
GP1BA	Glycoprotein Ib Platelet Subunit alpha	Chr. 17
GP1BB	Glycoprotein Ib Platelet Subunit beta	Chr. 22
GP6	Glycoprotein VI Platelet	Chr. 19
GP9	Glycoprotein IX Platelet	Chr. 3
HRG	Histidine Rich Glycoprotein	Chr. 3
ITGA2B	Integrin Subunit alpha 2b	Chr. 17
ITGB3	Integrin Subunit beta 3	Chr. 17
KLKB1	Kallikrein B1	Chr. 4
KNG1	Kininogen	Chr. 3
LMAN1	Lectin, Mannose Binding 1	Chr. 18
MCFD2	Multiple Coagulation Factor Deficiency 2, ER Cargo Receptor Complex Subunit	Chr. 2
MPL	Thrombopoietin receptor	Chr.1
MYH9	Myosin, Heavy Chain 9, Non-Muscle	Chr. 22
NBEAL2	Neurobeachin-Like Protein 2	Chr. 3

P2RY12	Purinergic Receptor P2Y12	Chr. 3
PLA2G4A	Phospholipase A2 Group IVA	Chr.1
PLAU	Plasminogen Activator, Urokinase	Chr. 10
PLG	Plasminogen	Chr. 6
PROC	Protein C	Chr. 2
PROCR	Endothelial Protein C Receptor	Chr. 20
PROS1	Protein S	Chr. 3
PROZ	Protein Z	Chr. 13
SERPINC1	Antithrombin	Chr.1
SERPINE1	Plasminogen Activator Inhibitor-1	Chr. 7
SLC44A	Solute Carrier Family 44 Member 2	Chr. 19
TBXA2R	Thromboxane A2 Receptor	Chr. 7
TBXAS1	Thromboxane A Synthase 1	Chr. 19
THBD	Thrombomodulin	Chr. 20
THPO	Thrombopoietin	Chr. 3
TUBB1	Tubulin Beta 1 Class IV	Chr. 20
VKORC1	Vitamin K Epoxide Reductase Complex, Subunit 1	Chr. 16
VWF	Von Willebrand Factor	Chr. 12
WAS	WASP Actin Nucleation Promoting Factor	Chr. X

Supplemental Table S2. Significant variants identified in patients with a history of cerebral vein thrombosis (CVT). SNP = single nucleotide polymorphism; Cat. = categorization of the variants as described in methods; Alt. Allele = number of alternative alleles; Sample Size = number of all alleles analyzed; Frequency = relative frequency of the alternative allele; p-value = resulting p-value of the test for proportions analyzing CVT vs. NCBI, p cor. = p-value after correction for multiple testing. CVT = data collected in the study group of 58 patients; NCBI (National Center for Biotechnology Information) = data collected from NCBI database. Variants with a frequency in NCBI database = 0 are indicated by light grey letters. A light grey background highlights variants significant after multiple testing (p-value < 0.05) and with a frequency in NCBI database > 0, but with a rare allele frequency in the study cohort (Alt. Allele < 6). White letters on black background highlight variants that are significant after multiple testing and show a frequency > 5% (Alt. Allele > 6) within the study cohort.

			CVT			NCBI				
**Gene**	**SNP**	**Cat.**	**Alt. Allele**	**Sample Size**	**Frequency**	**Alt. Allele**	**Sample Size**	**Frequency**	**p-value**	**p-value cor.**
ACTN1	rs74446338	IV	1	116	0.00862	0	9818	0	5.47E−06	4.61E−03
ACTN1	rs548666468	IV	3	116	0.02586	29	14336	0.00202	8.49E−06	7.16E−03
ACTN1	rs148344567	IV	1	116	0.00862	3	26434	0.00011	2.12E−04	1.79E−01
ACTN1	rs115674827	IV	1	116	0.00862	2	15928	0.00013	1.29E−03	>1
ACTN1	rs372305135	IV	1	116	0.00862	3	15878	0.00019	5.62E−03	>1
ACTN1	rs141539823	IV	1	116	0.00862	9	26722	0.00034	2.84E−02	>1
ACVRL1	rs1455796139	IV	1	116	0.00862	0	9690	0	6.32E−06	5.33E−03
ACVRL1	rs374020751	IV	1	116	0.00862	13	37110	0.00035	2.85E−02	>1
ADAMTS13	rs367887198	IV	1	116	0.00862	0	25120	0	2.41E−13	2.03E−10
ADAMTS13	rs28446901	IV	41	116	0.35345	2144	14192	0.15107	3.52E−09	2.97E−06
ADAMTS13	rs782206311	IV	1	116	0.00862	1	25064	0.00004	3.03E−07	2.55E−04
ADAMTS13	rs1554796703	IV	1	116	0.00862	0	9690	0	6.32E−06	5.33E−03
ADAMTS13	rs2073932	IV	48	116	0.41379	13568	23926	0.56708	1.24E−03	>1
ADAMTS13	rs739469	II	48	116	0.41379	8908	16936	0.52598	2.04E−02	>1
ANKRD26	rs1417891429	IV	1	116	0.00862	0	7618	0	6.59E−05	5.56E−02
ANKRD26	rs193178384	IV	1	116	0.00862	4	32782	0.00012	2.47E−04	2.08E−01
ANKRD26	rs66585308	IV	12	116	0.10345	470	13004	0.03614	3.32E−04	2.80E−01
ANKRD26	rs201149741	IV	1	116	0.00862	5	32646	0.00015	8.94E−04	7.53E−01
ANKRD26	rs192171184	IV	1	116	0.00862	2	14270	0.00014	2.12E−03	>1
ANKRD26	rs11015455	IV	18	116	0.15517	1117	12404	0.09005	2.33E−02	>1
CPB2	rs3742266	IV	6	116	0.05172	17526	130510	0.134289	1.35E−02	>1
ENG	rs370257876	III	1	116	0.00862	6	20372	0.00029	1.94E−02	>1
F10	rs549329855	IV	1	116	0.00862	1	14284	0.00007	1.29E−04	1.09E−01
F10	rs3211770	IV	5	116	0.04310	9260	77638	0.11927	1.70E−02	>1
F10	rs2026160	IV	71	116	0.61207	84985	119718	0.709877	2.66E−02	>1
F13A1	rs202215504	IV	1	116	0.00862	2	25799	0.00008	3.16E−05	2.66E−02

F13A1	rs7745910	IV	16	116	0.13793	915	12636	0.07241	1.17E−02	>1
F13B	rs5996	IV	58	116	0.50000	3990	11316	0.3526	1.35E−03	>1
F13B	rs201706582	IV	1	116	0.00862	1	9690	0.0001	1.68E−03	>1
F2	rs117682331	IV	3	116	0.02586	73	20214	0.00361	1.61E−03	>1
F2	rs377291293	IV	1	116	0.00862	3	14152	0.00021	8.96E−03	>1
F5	rs1227193883	IV	1	116	0.00862	1	134850	0.000007	3.23E−34	2.72E−31
F5	rs1384690038	II	1	116	0.00862	1	26588	0.00004	2.02E−07	1.70E−04
F5	rs752741472	IV	1	116	0.00862	0	9690	0	6.32E−06	5.33E−03
F5	rs755294580	IV	1	116	0.00862	2	26588	0.00008	2.88E−05	2.43E−02
F5	rs6026	II	1	116	0.00862	4	41612	0.0001	4.90E−05	4.13E−02
F5	rs6020	II	5	116	0.04310	2744	252178	0.010881	3.79E−03	>1
F5	rs80030498	IV	1	116	0.00862	4	15936	0.00025	1.41E−02	>1
F5	rs6022	III	30	116	0.25862	33508	192092	0.174437	2.35E−02	>1
F5	rs6029	III	30	116	0.25862	43915	244850	0.179355	3.54E−02	>1
F7	rs747200317	IV	1	116	0.00862	0	6962	0	1.39E−04	1.17E−01
F7	rs778654895	IV	1	116	0.00862	3	9690	0.0003	3.37E−02	>1
F7	rs2774033	IV	16	116	0.13793	2418	30214	0.08003	3.40E−02	>1
F8	rs146581224	IV	1	116	0.00862	46	153112	0.0003	1.37E−02	>1
F8	rs1800292	III	3	116	0.02586	7150	75048	0.09527	1.70E−02	>1
F8	rs137852388	III	1	116	0.00862	4	14152	0.00028	2.16E−02	>1
FGA	rs2070011	III	57	116	0.49138	115833	184614	0.627433	3.35E−03	>1
FGA	rs6050	I	42	116	0.36207	9608	38782	0.24774	6.16E−03	>1
FGG	rs200696007	IV	1	116	0.00862	1	14152	0.00007	1.34E−04	1.13E−01
FLNA	rs782598729	IV	2	116	0.01724	0	9690	0	4.62E−22	3.89E−19
FLNA	rs782410017	IV	2	116	0.01724	3	14152	0.00021	3.08E−13	2.60E−10
FLNA	rs375910592	IV	1	116	0.00862	0	9690	0	6.32E−06	5.33E−03
FLNA	rs782726997	IV	1	116	0.00862	0	9690	0	6.32E−06	5.33E−03
FLNA	rs369957061	IV	1	116	0.00862	0	9690	0	6.32E−06	5.33E−03
FLNA	rs200796698	IV	1	116	0.00862	0	9682	0	6.38E−06	5.38E−03
FLNA	rs2070826	IV	17	116	0.14655	756	12942	0.05841	1.41E−04	1.18E−01
FLNA	rs201762017	IV	1	116	0.00862	21	119028	0.000176	1.05E−03	8.87E−01
FN1	rs769644869	IV	1	116	0.00862	0	26588	0	4.81E−14	4.06E−11
FN1	rs56380797	IV	49	116	0.42241	1794	9290	0.1931	1.31E−09	1.10E−06
FN1	rs150990682	IV	1	116	0.00862	2	37110	0.00005	1.84E−07	1.55E−04
FN1	rs776072825	IV	1	116	0.00862	1	23860	0.00004	4.26E−07	3.59E−04
FN1	rs35343655	III	45	116	0.38793	5768	27622	0.20882	3.92E−06	3.31E−03
FN1	rs781267501	IV	1	116	0.00862	0	9690	0	6.32E−06	5.33E−03
FN1	rs373362388	IV	1	116	0.00862	3	26588	0.00011	2.10E−04	1.77E−01
FN1	rs370342980	IV	1	116	0.00862	4	26512	0.00015	1.13E−03	9.53E−01
FN1	rs56029730	IV	7	116	0.06034	259	13168	0.01967	5.42E−03	>1
FN1	rs1051959787	IV	1	116	0.00862	2	9824	0.0002	1.18E−02	>1

FN1	rs56137176	IV	43	116	0.37069	5377	20398	0.2636	1.23E−02	>1
FN1	rs56381916	IV	49	116	0.42241	1754	7286	0.2407	3.96E−02	>1
FN1	rs34923683	IV	5	116	0.04310	571	36910	0.01547	4.28E−02	>1
GGCX	rs7568458	IV	52	116	0.44828	3265	9458	0.3452	2.64E−02	>1
GP1BA	rs546035317	IV	1	116	0.00862	1	26588	0.00004	2.02E−07	1.70E−04
GP1BA	rs2243093	IV	7	116	0.06034	32460	256010	0.126792	4.43E−02	>1
GP6	rs201652983	IV	1	116	0.00862	0	9690	0	6.32E−06	5.33E−03
GP6	rs376003141	IV	1	116	0.00862	0	9690	0	6.32E−06	5.33E−03
GP6	rs937978607	IV	1	116	0.00862	1	14152	0.00007	1.34E−04	1.13E−01
GP6	rs189534942	IV	6	116	0.05172	254	20348	0.01248	8.14E−04	6.87E−01
ITGA2B	rs146829521	IV	2	116	0.01724	5	14278	0.00035	1.03E−09	8.65E−07
ITGA2B	rs762724640	IV	1	116	0.00862	0	6962	0	1.39E−04	1.17E−01
ITGA2B	rs146382550	III	1	116	0.00862	28	169914	0.000165	6.42E−04	5.41E−01
ITGA2B	rs377288289	IV	1	116	0.00862	4	14286	0.00028	2.14E−02	>1
ITGB3	rs375647456	IV	2	116	0.01724	1	15752	0.00006	4.78E−24	4.03E−21
ITGB3	rs567581451	IV	2	116	0.01724	5	12076	0.00041	2.10E−08	1.77E−05
ITGB3	rs36080296	III	2	116	0.01724	464	190484	0.002436	2.22E−02	>1
KLKB1	rs148277206	IV	2	116	0.01724	83	169946	0.000488	2.04E−09	1.72E−06
KLKB1	rs2292423	IV	52	116	0.44828	5799	17672	0.32815	8.15E−03	>1
KNG1	rs11057	IV	1	116	0.00862	1	20316	0.00005	4.65E−06	3.92E−03
KNG1	rs577060476	IV	1	116	0.00862	0	9690	0	6.32E−06	5.33E−03
KNG1	rs368847123	IV	1	116	0.00862	3	32650	0.00009	3.73E−05	3.14E−02
LMAN1	rs771859168	IV	1	116	0.00862	0	9686	0	6.35E−06	5.35E−03
LMAN1	rs8095245	IV	28	116	0.24138	1625	10748	0.15119	1.05E−02	>1
MYH9	rs150539764	IV	1	116	0.00862	0	9688	0	6.33E−06	5.34E−03
MYH9	rs76330062	IV	8	116	0.06897	396	19392	0.02042	8.57E−04	7.23E−01
MYH9	rs710181	III	108	116	0.93103	137766	141908	0.970812	2.34E−02	>1
NBEAL2	rs11396344	IV	2	116	0.01724	12042	12078	0.99702	0.00E+00	0.00E+00
NBEAL2	rs779996125	IV	1	116	0.00862	0	15752	0	7.30E−09	6.16E−06
NBEAL2	rs200100160	IV	2	116	0.01724	16	32768	0.00049	1.18E−08	9.94E−06
NBEAL2	rs371752213	IV	1	116	0.00862	0	9690	0	6.32E−06	5.33E−03
NBEAL2	rs927922270	IV	1	116	0.00862	0	9690	0	6.32E−06	5.33E−03
NBEAL2	rs369490668	IV	1	116	0.00862	0	9690	0	6.32E−06	5.33E−03
NBEAL2	rs377667217	IV	1	116	0.00862	0	9690	0	6.32E−06	5.33E−03
NBEAL2	rs11396344	IV	113	116	0.97414	12042	12078	0.99702	4.35E−04	3.66E−01
NBEAL2	rs13081418	IV	49	116	0.42241	9966	30814	0.32342	2.96E−02	>1
P2RY12	rs1030232393	IV	2	116	0.01724	10	14152	0.00071	6.79E−06	5.73E−03
PLAU	rs2227564	III	73	116	0.62931	155743	201354	0.773479	3.22E−04	2.71E−01
PLAU	rs2227566	III	55	116	0.47414	89750	153946	0.582997	2.24E−02	>1
PLG	rs1016440595	IV	1	116	0.00862	2	26588	0.00008	2.88E−05	2.43E−02
PLG	rs144424879	IV	1	116	0.00862	1	15752	0.00006	4.33E−05	3.65E−02

PLG	rs889957249	IV	1	116	0.00862	0	6962	0	1.39E−04	1.17E−01
PLG	rs370893923	IV	1	116	0.00862	1	9824	0.0001	1.61E−03	>1
PROC	rs777364878	II	1	116	0.00862	4	32650	0.00012	2.48E−04	2.09E−01
PROCR	rs199638108	IV	1	116	0.00862	11	96818	0.00011	3.80E−05	3.20E−02
PROCR	rs945960	IV	66	116	0.56897	4011	8552	0.469	4.05E−02	>1
PROS1	rs775736913	IV	1	116	0.00862	0	9690	0	6.32E−06	5.33E−03
PROZ	rs934295767	IV	5	116	0.04310	0	3232	0	3.38E−26	2.85E−23
PROZ	rs1319808070	IV	5	116	0.04310	7	7618	0.0009	4.89E−25	4.12E−22
PROZ	rs986708698	IV	2	116	0.01724	0	2224	0	4.98E−06	4.20E−03
PROZ	rs955281823	IV	1	116	0.00862	0	4800	0	1.70E−03	>1
PROZ	rs1321706517	IV	1	116	0.00862	1	7618	0.0001	3.33E−03	>1
PROZ	rs45596441	IV	9	116	0.07759	900	30152	0.02985	6.26E−03	>1
PROZ	rs2480948	IV	27	116	0.23276	1786	12034	0.14841	1.61E−02	>1
SERPINC1	rs5877	II	58	116	0.50000	53942	151314	0.35649	1.76E−03	>1
SERPINC1	rs5878	II	57	116	0.49138	55131	153004	0.360324	4.48E−03	>1
SLC44A2	rs1181268521	IV	1	116	0.00862	0	6962	0	1.39E−04	1.17E−01
SLC44A2	rs375469547	IV	1	116	0.00862	9	37110	0.00024	7.48E−03	>1
TBXA2R	rs1193074174	IV	1	116	0.00862	0	9690	0	6.32E−06	5.33E−03
TBXA2R	rs201199706	IV	1	116	0.00862	10	32784	0.00031	2.00E−02	>1
TBXA2R	rs55753816	IV	20	116	0.17241	1523	14286	0.10661	3.30E−02	>1
TBXAS1	rs140774405	IV	1	116	0.00862	32	174408	0.000183	1.22E−03	>1
TBXAS1	rs6139	IV	1	116	0.00862	1303	14336	0.09089	3.53E−03	>1
THPO	rs1163856631	IV	1	116	0.00862	0	9690	0	6.32E−06	5.33E−03
THPO	rs1012887779	IV	1	116	0.00862	0	7618	0	6.59E−05	5.56E−02
TUBB1	rs144336677	IV	5	116	0.04310	137	14414	0.0095	1.42E−03	>1
VWF	rs374459416	IV	1	116	0.00862	1	15752	0.00006	4.33E−05	3.65E−02
VWF	rs41276736	III	5	116	0.04310	397	37114	0.0107	3.49E−03	>1
VWF	rs3741906	IV	18	116	0.15517	19325	230164	0.083962	9.41E−03	>1
VWF	rs2286937	IV	17	116	0.14655	1199	14982	0.08003	1.42E−02	>1
VWF	rs2070885	IV	17	116	0.14655	1157	14336	0.08071	1.58E−02	>1
VWF	rs2286938	IV	17	116	0.14655	1664	20348	0.08178	1.81E−02	>1
VWF	rs177702	III	83	116	0.71552	15881	19770	0.80329	2.43E−02	>1
VWF	rs1800383	III	14	116	0.12069	2492	35830	0.06955	4.81E−02	>1
